# Does board diversity reduce the probability of financial distress? Evidence from Chinese firms

**DOI:** 10.3389/fpsyg.2022.976345

**Published:** 2022-09-14

**Authors:** Shahid Ali, Shoukat Ali, Junfeng Jiang, Martina Hedvicakova, Ghulam Murtaza

**Affiliations:** ^1^School of Management Science and Engineering, Nanjing University of Information Science and Technology, Nanjing, Jiangsu, China; ^2^Department of Commerce, The Islamia University of Bahawalpur, Bahawalpur, Punjab, Pakistan; ^3^Department of Economics, Faculty of Informatics and Management, University of Hradec Králové, Hradec Králové, Czechia

**Keywords:** board diversity, financial distress, State-Owned Enterprises, Non-State-Owned Enterprises, China

## Abstract

This paper empirically investigates the impact of cognitive board diversity in education, expertise, and tenure facets on financial distress likelihood in the emerging economy of China. This study examines how this relationship varies across State-Owned Enterprises (SOEs) and Non-State-Owned Enterprises (NSOEs). Paper argues that the Chinese stock market, as a typical emerging market, is an excellent laboratory for studying the impact of board diversity on the probability of financial distress. Its underdeveloped financial system and inadequate investor protection leave firms unprotected from financial hardship. A sample of 12,366 observations from 1,374 firms from 2010 to 2018 shows that cognitive diversity qualities are positively linked with Z-score, implying that directors with different educational backgrounds, financial skills, and tenures can assist in reducing the probability of financial distress. Cognitive board diversity reduces the likelihood of financial distress in SOEs and NSOEs. However, tenure diversity is insignificant in all cases. Furthermore, the robustness model “two-step system Generalized Methods of Moments (GMM)” demonstrated a positive association between educational diversity, financial expertise, and financial distress scores. The results have significant implications for researchers, managers, investors, regulators, and policymakers.

## Introduction

Predicting financial distress is essential in businesses such as banking, investment, and manufacturing (Ali et al., 2021a). A firm’s financial uncertainty imposes considerable costs, such as reduced revenues and high legal and financing costs (Zhou, 2019). In the recent financial crisis, many firms filed for bankruptcy due to financial distress (Li and Zhong, 2013). Therefore, it works by investigating the ways to reduce financial distress likelihood. Although researchers, since the sixties, started to develop financial distress detection models (Beaver, 1966; Altman, 1968), most of them considered either financial (Altman, 1968; Taffler, 1983) or market data (Zmijewski, 1984; Almamy et al., 2016) as a predictor of financial distress. Moreover, limited studies have examined corporate governance implications for financial distress likelihood (Darrat et al., 2016; Udin et al., 2017). Little is investigated about the boards’ ex-ante behavior behind the event of financial distress. Thus, this study probes the effect of corporate board diversity on the probability of financial distress.

Theoretically, financial distress is usually caused by wrong financial decisions (Al-Hadi et al., 2017; Bhaskar et al., 2017), weak internal control systems, poor managerial policies, insufficient information disclosure, and failure to recognize stakeholder rights (Shahwan, 2015; Maina and Sakwa, 2017). These unfavorable situations arise because of agency problems. Therefore, expanding our understanding of the board’s role in failed firms is important (Adams and Ferreira, 2009). Diverse boards can control the entrenchment behavior of management by linking Chief Executive Officers (C.E.O.) turnover and compensation to the firm’s performance (Usman et al., 2018; Khan et al., 2020), improving monitoring intensity (Ararat et al., 2015; Hemdan et al., 2021), oversight of investment (Harjoto et al., 2018), and reducing the availability of free cash flow to managers through lowering capital structure and paying a high dividend (Bernile et al., 2018). Resultantly, it helps reduce the probability of financial hardship (Yousaf et al., 2021).

Research on the impact of board composition on financial distress is limited. Studies have tended to measure financial distress exclusively by gender (Kristanti et al., 2016; Mittal and Lavina, 2018). However, most recent studies have concluded that cognitive diversity facets (education, experience, and tenure) are more related to firm outcomes (Bernile et al., 2018; Harjoto et al., 2018) and are more valued by investors (De-La-Hoz et al., 2018). For example, Harjoto et al. (2018) concluded that studies that measured board diversity by using the only demographic board diversity attributes overlooked the importance of various dimensions in the board. Because cognitive attributes are more associated with the processing and assimilation of information than demographic attributes (Harjoto et al., 2018). For example, educational level is believed to shape how board members analyze things (Kagzi and Guha, 2018), while board members with long tenure are more informed about managerial behaviors, firm resources, organizational culture, and system. Then they make rational decisions (Bell et al., 2011). Besides, board members with financial experience critically analyze a firm’s financial matters and effectively contribute toward financial decisions related to investments, capital structure, and dividend policy (Sarwar et al., 2018). This study targets to make public the influence of cognitive board diversity attributes on the probability of financial distress. This research will let researchers, shareholders, and policymakers know: (i) the effect of previously unexplored diversity facets such as education, expertise, and tenure on financial distress likelihood, and (ii) how this relationship exists through State-Owned Enterprises (SOEs) and Non-State-Owned Enterprises (NSOEs).

Consequently, this study used Chinese firms to test the proposed hypothesis. The likelihood of a Chinese company’s bankruptcy has increased, as indicated in Moody’s sovereign reduced Chinese debt rating (Al-Hadi et al., 2017). To limit the probability of financial distress, the diverse board can effectively monitor China, where there are weak institutions and lower investor protection (Talavera et al., 2018). Besides, Chinese firms include directors with diverse backgrounds to enhance the board’s efficiency (McGuinness et al., 2017; Talavera et al., 2018; Guizani and Abdalkrim, 2022). On the other hand, regulatory and legislative bodies in China have neither legislated nor required directors’ diversity on boards. Therefore, to guide management, shareholders, and regulators in China, it is vital to probe the starring role of board diversity in predicting financial distress likelihood.

Considering the gap in the empirical research, the objectives of this study are as follows: To determine the effects of board diversity on the probability of financial distress and to test the moderating effect of state ownership on the link between board diversity and the probability of financial distress. The sample consists of 12,366 firm-year observations from 1,374 non-financial firms in the Chinese equity markets from 2010 to 2018. Distinctly, the fixed-effects of the panel regression method are employed to examine the relationships. The results revealed that board diversity in education, financial expertise, and tenure diversity reduces the probability of financial distress. These results are valid irrespective of the type of enterprise. Further, the robust models verified the results and applied two-stage GMM to resolve the possible endogenous problem.

The study augments the present writings in three ways. First, limited studies investigating the connection between directors’ diversity on the board and financial distress have investigated gender as an important board diversity attribute (Francoeur et al., 2008; Platt and Platt, 2012; Mittal and Lavina, 2018; Zhou, 2019). In a systematic literature review, Khatib et al. (2021) emphasized the need to explore board diversity attributes rather than focusing only on the board gender diversity. Our research, however, considers three cognitive diversity facets discussed above. As per researchers’ knowledge, this is one of the earliest researches to collectively integrate cognitive board diversity attributes into financial distress prediction models. Second, this research expands the viewpoint of agency theory using Altman et al. (2007)’s Z-score model designed explicitly for Chinese firms to offer robust evidence. Consistent with agency theory, the results of this research indicate that cognitive board diversity attributes, including education, expertise, and tenure reduces the financial distress likelihood in China. Third, this study unveils the importance of cognitive diversity attributes in reducing financial distress for both SOEs and NSOEs. The results enhance our understanding by concluding that board diversity effectively reduces the financial distress likelihood irrespective of the enterprise form.

The paper will follow the structure as the next section surveys the evolution of literature and hypotheses, while the following sections outline the research design. Section four offers the results and its discussion before the final section of the paper concludes.

## Literature review and hypothesis development

### Educational diversity and financial distress

Educational level is a vital diversity attribute that affects the cognitive abilities of the board to evaluate alternatives for strategic decisions and repel management from using the firm’s properties for self-interest sacrificing shareholders’ interests. Directors’ educational level indicates their cognitive orientation, knowledge, and skills (Hambrick and Mason, 1984). Firms founded and managed by highly educated executives tend to outperform those operated by ill-educated executives (Kristanti et al., 2016). Likewise, Gottesman and Morey (2006) also reinforced that the educational background of top management is an imperative intellectual capital for firms. Consistently, literature revealed that education increases directors’ ability to embrace new ideas and understand strategic matters (Post et al., 2011). Drawing on the agency theory, educational diversity improves directors’ ability to control managerial entrenchment behavior by evaluating strategic decisions (Hitt and Tyler, 1991), aligning executives’ pay with firm performance, and controlling earning management. Hambrick and Mason (1984) posit that higher education progresses directors’ ability to route information, enhance open-mindedness, and accept change. According to the social identity theory of Tajfel (1978), board members with the same attributes will classify themselves into in-group and out-group categories (Veltrop et al., 2015). This social classification into in-group and out-group hinders communication and group working (Chen et al., 2016). According to social identity theory, board members with different levels of education may categorize into groups (Kagzi and Guha, 2018). Out-group members are perceived as insincere and less cooperative than in-group fellows (Brewer, 1979), leading to group bias. According to Ali et al. (2014), boards with diverse educational levels will involve constrained communication.

A plethora of literature has established that board members’ educational qualification is linked to firm performance (Kagzi and Guha, 2018; Cumming and Leung, 2021), but the findings are mixed and inconclusive. Some studies confirm positive relationships, while others, though limited, found a negative or no connection. For example, Ujunwa et al. (2012) uncovered PhD directors’ impact on firms’ financial performance using 122 listed Nigerian companies from 1991 to 2008 and found a direct effect of PhD directors on a firm’s economic performance. Kim and Lim (2010) studied South Korean Firms to observe the sway of directors’ educational diversity on firm value and established that the educational diversity of directors could improve firm valuation. Cheng et al. (2010) researched China to observe the influence of the board chairman’s qualification on the firms’ accounting performance and revealed that educational level is positively connected with earnings per share and ROA. Likewise, Darmadi (2013) supports that directors’ educational level positively affects a firm’s value.

In contrast, Khan et al. (2019) determined that educational diversity in Pakistan is inversely associated with corporate social responsibility (CSR). However, Bernile et al. (2018) used United States firms as a sample and found an immaterial effect of board educational diversity on firm risk. Likewise, in India, Kagzi and Guha (2018) communicated a negative link between the level of education and a firm’s Tobin’s Q, inferring that investors perceive board educational diversity as a possible source of conflict. Moreover, Harjoto et al. (2018) also find an insignificant impact of board education diversity on investment oversight. Hence, the study hypothesized:

*Hypothesis 1: Boards*’ *educational diversity reduces the probability of financial distress.*

### Financial expertise and financial distress

The corporate scandals, including Enron, Tyco, and World Com, attracted regulators’ attention to include financial experts on the corporate boards (Güner et al., 2008). Given the importance of accounting and financial education, SOX (2002) requires selecting directors with financial backgrounds (Dhaliwal et al., 2010). A financial expert with a financial background is integral to the internal corporate governance mechanism to control and oversee the managers’ self-serving behavior (Strebel and Lu, 2010). For instance, Güner et al. (2008) argued that financial education and experience help acquire information regarding complex financial transactions at a lower cost and professionally work for shareholders’ interests (Harris and Raviv, 2008). Motivated directors without financial expertise may not properly assess a company’s finances (Gore et al., 2011). Therefore, you need financial skills to grasp the benefits and drawbacks of financial choices (Young, 2004).

The empirical literature has revealed value in having directors with financial expertise. Zalata et al. (2018) concluded that directors’ competence is an effective tool to reduce earning management in firms. CEOs with MBA can make aggressive choices such as larger debt ratio, lower dividend payments, and more capital expenditure, and outperform CEOs without an MBA (Bertrand and Schoar, 2003). Likewise, Manzaneque et al. (2016) reported that directors having financial education positively affects a firm’s information disclosure. Likewise, Jensen and Zajac (2004) concluded that financially expert directors positively affect a firm’s acquisition and diversification. The good market reaction to the nomination of financial experts to audit committees, as stated by DeFond et al. (2005), suggests that the market values financial skills to increase the audit committee’s capacity to assure first-rate financial reporting. However, previous literature has far and wide probed the influence of financial expertise attributed to the board of directors on financial distress likelihood. This is among the earliest studies to examine board financial expertise and financial distress in China’s growing economy. So, this research linked board financial expertise to financial distress as given below:

*Hypothesis 2: Board financial expertise reduces the probability of financial distress.*

### Board tenure diversity and financial distress

Tenure reveals directors’ experience as board members and familiarity with the firm’s resources, policies, and operations. Vafeas (2003) developed two alternative hypotheses regarding directors’ tenure (i) management friendly and (ii) expertise hypothesis. The expertise hypothesis states that a director’s length of service at a company is a good indicator of the director’s familiarity with the company’s operations and workplace culture. Long-tenured directors have more competency and experience than short-tenured directors. For example, long tenure members know the firm’s strategies and managerial practices and can oversee management effectively (Kesner, 1988). Kim et al. (2014) suggested that the tenure of a director enhances the director’s capability to perform their advising and monitoring role to nurture firm value because directors having long tenure are more willing to attend board and committee meetings, more likely to accept committee assignments, have more understanding about firm’s strategic policies. They argue that long tenure board members control agency problems by lowering CEO rent extraction leading to better investment and acquisition policy and higher firm operating performance.

While, management friendly hypothesis reveals that long-serving directors monitor less effectively as they tend to have friendships with the managers, and in turn, longer tenure affects corporate performance adversely (Finkelstein and Hambrick, 1989; Vafeas, 2003). Hambrick and Fukutomi (1991) contended that executives, early in their tenure, are energetic to acquire the maximum possible knowledge about the firm and its operations. But over time, as tenure prolongs, they think the current management model is appropriate to deal with challenges faced by firms and lack motivation to get up-to-date knowledge about changes in operational market demand. This attitude of long-tenured senior executives will deteriorate the firm’s ability to react to changes in the market and may deteriorate the firm’s performance. In this line, Bernile et al. (2018) revealed that a large fraction of long-tenured board members reduces board effectiveness to reduce firm risk because long tenure results in the emergence of group thinking or bondage to the CEO hampering the board’s d’s monitoring role.

In contrast, board members who served for a shorter period are less prone to form attachments to management but may have more difficulty expressing an effective critical perspective. For example, Kipkirong Tarus and Aime (2014) found that young directors are more engaged in strategic decision-making and risky ventures due to their risk-taking behavior. Thus, by considering benefits and costs attached to short and long tenure, tenure diversity is anticipated to generate a balanced effect on a firm’s accounting and market performance. Contemporary research on board diversity and business value find varied results. For example, Khan et al. (2019) used 86 firms’ samples from the Pakistan Stock Exchange (PSX) to investigate the bearing of tenure diversity on CSR and found a strong correlation between tenure diversity and CSR in Pakistan, suggesting that tenure increase creativity as well as expertise in monitoring.

In comparison, Bernile et al. (2018) and Harjoto et al. (2018) found no influence of board diversity on risk and investment oversight. Despite growing learning on the economic implications of tenure diversity, literature on board tenure diversity and financial distress is sparse (Kanadlı et al., 2020). This study inspects Chinese enterprises to fill a literature vacuum. This study advocates an association between board tenure diversity and the probability of financial distress.

*Hypothesis 3: Board tenure diversity reduces the probability of financial distress.*

## Research methodology

### Sample and data source

From 2010 to 2018, we used data from Chinese firms listed on the Shanghai and Shenzhen stock markets. This study uses data from 2010 since the Global Financial Crisis (GFC) of 2007–2008 is well-known, and financial hardship might lead to misleading results for GFC years. Data for research variables are obtained from the Chinese Stock Market and Accounting Research (CSMAR) database. Companies listed on the Shanghai and Shenzhen stock exchanges were included in the study during the sample period.

Furthermore, due to their distinct reporting and regulatory patterns, to ensure comparability of results, we exclude banks and real estate companies from the initial sample (Ali et al., 2021b). A total of 12,366 firm-year observations from 1,374 non-financial firms were included in the final sample. In addition, all variables in this analysis have been winsorized at 5 and 95% to lessen the impact of outliers. Finally, compared to past studies, we collected our sample from China’s 14 distinct industries (McGuinness et al., 2017; Shahab et al., 2018). Table 1 shows the industrial distribution of the entire sample. According to this breakdown, most Chinese listed enterprises manufacture (61.14). After the manufacturing business, the wholesale and retail sectors (8.37) and electricity, heat, gas, and water production (6.04) represent the most represented sectors.

**TABLE 1:** Industrial distribution of data.

Industry	*n*	%
Accommodation and catering	81	0.66
Complex industries	207	1.67
Construction industry	72	0.58
Culture, sports, and entertainment	189	1.53
Farming, forestry, animal husbandry, and fishery	216	1.75
Health and social work	243	1.97
Information technology	612	4.95
Learning and business services	225	1.82
Manufacturing industry	7,560	61.14
Mining sector	513	4.15
Production and supply of power, heat, gas, and water	747	6.04
Transportation, storage, and postal services	594	4.8
Water, environment, and public facilities management industry	72	0.58
Wholesale and retailing	1,035	8.37
Total	12,366	100

### Variables

#### Financial distress

This study’s dependent variable is the probability of financial stress. China Specific Altman Z Score established by Altman et al. (2007) is derived as Z-Score = 6.56*(Sales/Total Assets) + 3.26*(Retained Earnings/Total Assets) + 6.72*(EBIT/Total Asset) + 1.05*(Book Value of Equity/Total Asset) to evaluate financial distress likelihood (Zhang et al., 2010; Shahab et al., 2018; Ali et al., 2021b).

#### Board diversity

Directors’ education, experience, and tenure were independent factors in this investigation. The Blau index (2000) is used to quantify these diversity traits in this study as follows:


$$D=1-\Sigma \,p\,i^{2}$$

I signify the number of categories where p is the proportion of each group. Index 1 indicates perfect demographic heterogeneity, whereas index 0 indicates homogeneity. The maximum diversity index (D) value grows with category count. If there are four equal-sized categories, the diversity index will be 0.75 [1 (0.252 + 0.252 + 0.252)]. The value of the diversity index will rise with the increase in the number of categories. The diversity index (D) was used to quantify cognitive diversity traits such as education, financial competence, and tenure. In this study, financial expertise diversity is measured as a categorical variable using two cates: having or not having financial experts on board. Lastly, tenure diversity is measured using six categories: less than or equal to 3 years, 4 to 6 years, 7 to 9 years, 10 to 12 years, 13 to 15 years, and 16 years and above.

#### Control variables

This study includes six control variables to minimize misleading connections between variables and model specification errors (Udin et al., 2017). Following previous literature, the control variables include company size, leverage, liquidity, board independence, board size, and return on assets (Zhou, 2019; Bravo-Urquiza and Moreno-Ureba, 2021; Ali et al., 2021a).

Financial hardship increases when F Leverage, F.S. expands (Nugrahanti et al., 2020; Ud-Din et al., 2020) and declines as F Liquidity, B.I., BS, ROA grows (Zhou, 2019; Ud-Din et al., 2020). Table 2 contains definitions for these variables.

**TABLE 2:** Variable descriptions.

Variables	Symbols	Measurement of variables
**Dependent variable**		
Financial distress	F.D. Score	Altman Z_Score calculated as Z-Score = 6.56*X_1_ + 3.26*X_2_ + 6.72*X_3_ + 1.05*X_4_ Where, X_1_ = Sales/Total Assets, X_2_ = Retained Earnings/Total Assets, X_3_ = EBIT/Total Assets, X_4_ = Book Value of Equity/Total Assets (Altman et al., 2007; Zhang et al., 2010; Ali et al., 2021a).
**Independent variables**		
Education diversity	Education_D	Index of education variety uses five categories: Technical secondary school and lower, Associate degree, Bachelor, Master, and Ph.D. indicated by 1,2,3,4,5, respectively (Ararat et al., 2015; Ali et al., 2021a).
Expertise diversity	Expertise_D	Index determined for directors with or without prior experience in financial matters (Bernile et al., 2018; Ali et al., 2021a).
Tenure diversity	Tenure_D	Calculating the index of tenure diversity involves using the following six categories: 3 years or less in age,4–6, 7–9, 10–12, 13–15, and more than 15 years (Harjoto et al., 2018; Bhat et al., 2020; Ali et al., 2021b).
**Control variables**		
Firm size	F.S.	The natural log of a company’s total assets is used to calculate firm size (Usman et al., 2022).
Firm leverage	F_Leverage	Total liabilities to total assets ratio (Sarwar et al., 2020; Ain et al., 2022).
Firm liquidity	F_Liquidity	Current assets to current liabilities ratio (Yeh, 2018; Ullah et al., 2018).
Return on assets	ROA	Net income-to-assets ratio (Yeh, 2018; Ullah et al., 2018; Ain et al., 2022).
Board size	BS	Total directors on the firm’s board (Bhat et al., 2020; Ain et al., 2022).
Board independence	B.I.	Calculate by dividing independent directors by overall directors (Yeh, 2018; Bhat et al., 2020; Usman et al., 2022).

### Econometric technique

Omitted variables that impact director selection and financial distress might lead to erroneous correlations. Progressive firms may have strong governance resulting in low chances of financial distress. The Hausman test decides between fixed and random effects to avoid omitted variable bias. Governance researchers commonly employ fixed or random effect regression to eliminate omitted variable effects (e.g., Adams and Ferreira, 2009; Manzaneque et al., 2016; Farag and Mallin, 2017; Yeh, 2018; Naseem et al., 2020). Corporate success, board diversity, and unobservable variability may have a reverse causal connection, presenting an endogeneity dilemma. The GMM estimator effectively handles endogeneity problems (Sarwar et al., 2018; Ullah et al., 2018; Ali et al., 2021a,b). The GMM avoids endogeneity by adding lagged dependent variable instruments (Arellano and Bond, 1991; Ullah et al., 2018). Hence, the model prevents data loss (Ullah et al., 2018). The study estimates our models using the dynamic two-step GMM model, eliminating potential endogeneity and reverse causality difficulties (Bhat et al., 2020; Sarwar et al., 2020). The study further separated the sample into SOEs and NSOEs to explore the link between cognitive board diversity and the probability of financial distress across different types of ownership.

The following model is used to test our proposed hypotheses.


$$F\,D_{i,t}=\beta_{0}+\beta_{2}\,D\,i\,v\,e\,r\,s\,i\,t\,y_{i,t}+\Sigma \,\beta_{3}\,C\,o\,n\,t\,r\,o\,l_{i,t}+\varepsilon_{i,t}$$

The dependent variable is F.D. (Financial Distress), whereas the independent variable is Diversity, revealing board diversity attributes such as D_Ten, D_Exp, and D_Edu. Furthermore, control includes firm size, firm leverage, liquidity, return on investment, board size, and board independence.

## Results and discussion

### Descriptive statistics

Descriptive data for sample firms in terms of mean, standard deviation, 5th percentile, median and 95th percentile for the study variables are shown in Table 3. This study’s dependent variable is financial distress (F.D.) measured using the Altman z-score. This Z-score categorizes firms into three groups: those that are stable (Z-scores greater than 0.90), those that are probably troubled (Z-scores between 0.5 and 0.90), and those that are in financial distress (Z-scores less than 0.5) (Altman et al., 2007; Zhang et al., 2010; Ali et al., 2021b).

**TABLE 3:** Descriptive statistics.

Variable	Obs	Mean	Std. Dev.	Min	Max	5th percentile	Median	95th percentile
Dependent variable								
FD	12,366	0.63	0.76	−1.27	1.52	−1.24	−0.10	1.49
Independent variables								
Education_D	12,366	0.08	0.05	0.00	0.61	0.00	0.10	0.15
Expertise_D	12,366	0.41	0.09	0.00	0.5	0.20	0.44	0.50
Tenure_D	12,366	0.12	0.07	0	0.45	0.00	0.10	0.15
Control variables								
FS	12,366	22.23	1.2	14.43	24.48	20.41	22.18	24.52
F_Lev	12,366	0.48	0.21	0.13	0.83	0.14	0.48	0.82
F_Liq	12,366	0.27	0.34	0.02	1.29	0.01	0.12	1.34
ROA	12,366	0.05	0.11	−0.07	0.45	−0.06	0.03	0.44
BS	12,366	2.18	0.17	1.8	2.5	1.79	2.20	2.48
BI	12,366	0.37	0.05	0.34	0.5	0.33	0.33	0.50

Please see Table 2 for variable definitions.

The F.D. score for Chinese enterprises is 0.63 on average, with a high of 1.52 and a minimum of −1.27. This indicates that most Chinese enterprises in our study area are in a potential distress zone. Board diversity is an independent variable, which includes three categories: education, experience, and tenure. The average Blau education diversity score is 0.08, indicating that education diversity in Chinese listed firms is minimal on average. On the other hand, expert diversity has an average of 0.41 and a median of 0.44, illustrating relatively higher financial experts on boards.

In comparison, tenure diversity is 0.12 on average. These descriptive reveal that average educational and tenure diversity are far lower than financial knowledge diversity. It implies that the average board of directors has higher financial knowledge but less tenure and education. The control variables, which include corporate size, leverage, liquidity, ROA, independent board, and board size for corporations, have a mean of 22.23, 0.48, 0.27, 0.05, 2.18, and 0.37, respectively.

The correlation matrix for the studied variables is shown in Table 4. Weak correlations indicate no multicollinearity. Board diversity attributes Edu D, Exp D, Ten D, and FD SCORE are associated with 0.03, 0.12, and 0.02, respectively (significant at 1 percent level). These relationships, on the whole, do not contradict our research assumptions. Furthermore, the independent and control variables have low correlation coefficients, indicating no issue of multicollinearity. The variables’ Variance Inflation Factors (VIFs) are also reported, with VIF values ranging from 1.01 to 1.52, far below the conventional threshold of 5.

**TABLE 4:** Variance inflation factor (VIF) and correlation matrix.

Variables	VIF	1	2	3	4	5	6	7	8	9	10
1 FD	–	1.00									
2 Education_D	1.02	0.03*	1.00								
3 Expertise_D	1.01	0.12*	−0.01	1.00							
4 Tenure_D	1.05	0.02*	0.06*	0.07*	1.00						
5 FS	1.42	−0.08*	−0.03*	0.04*	0.13*	1.00					
6 F_Lev	1.33	−0.09*	−0.09*	−0.02	−0.05*	0.28*	1.00				
7 F_Liq	1.52	0.12*	0.03*	−0.04*	−0.01	−0.45*	−0.45*	1.00			
8 ROA	1.06	0.15*	0.06*	−0.02	−0.06*	−0.07*	−0.18*	0.18*	1.00		
9 BS	1.39	−0.08*	0.03*	0.03*	0.01	0.22*	0.12*	−0.22*	−0.05*	1.00	
10 BI	1.3	0.06*	−0.01	0.03*	0.02	0.03*	−0.01	0.04*	0.01	−0.46*	1.00

*Significant at 5% level.

### Effect of board diversity on financial distress likelihood

To address the issue of omitted variable bias, the Hausman test decides between random effect and fixed-effect models. The Hausman test (chi^2^ = 502.60 and prob > chi^2^ = 0.000) rejected the null hypothesis of no association between residuals and explanatory variables and confirmed the fixed-effect regression’s applicability for sampled firms. Table 5 displays the findings of the fixed-effect model. This study employed the Blau diversity index to quantify models’ education, financial, and tenure board diversity. H1 suggests that educational variety reduces the likelihood of financial trouble. As predicted, education board diversity is directly associated with financial distress score (= 1.099, *p* 0.01). A high F.D. score indicates less financial distress and vice versa.

**TABLE 5:** Fixed effect regression results.

Dependent variables				

Variables	FD (1)	FD (2)	FD (3)	FD (4)
Education_D	1.0995***			0.9619***
	0.2075			0.21
Expertise_D		0.7419***		0.6789***
		0.7214		0.072
Tenure_D			0.9667***	0.8777***
			0.0919	0.0919
FS	−0.0205**	−0.0245**	−0.0456***	−0.0436***
	0.0087	0.0087	0.0089	0.0091
F_Leverage	0.2619***	−0.2451***	−0.2463***	−0.2056***
	0.4465	0.0445	0.0445	0.0444
F_Liqudity	0.1393***	0.1479***	0.1296***	0.1323***
	0.0276	0.0275	0.0275	0.0274
ROA	0.7149***	0.7265***	0.7639***	0.7444***
	0.0661	0.0658	0.0659	0.0656
BS	−0.2811***	−0.2674***	−0.2202**	−0.2235**
	0.706	0.0704	0.0706	0.0702
BI	−0.0219	0.01672	0.0042	0.0044
	0.2026	0.2018	0.2018	0.2008
Constant	1.0197***	0.8397**	1.4484***	1.0291***
	0.2854	0.2849	0.2848	0.2862
Year dummies	Yes	Yes	Yes	Yes
Observations	12,366	12,366	12,366	12,366
Adjusted R2	0.51	0.43	0.52	0.56
Number of companies	1,374	1,374	1,374	1,374

***p < 0.01, **p < 0.05.

In H2 and H3, financial knowledge and tenure board diversity are hypothesized to decrease the likelihood of financial distress. In Models 2 and 3 of Table 5, financial expertise and tenure board diversity are strongly correlated with financial distress scores as expected (= 0.742, *p* 0.01; = 0.967, *p* 0.01). The study found that enhancing board financial knowledge and tenure diversity can help organizations achieve greater financial performance and lower the chance of financial trouble. Furthermore, all three cognitive board diversity attributes are included collectively in model 4 of Table 5 to reveal the marginal effects of these board diversity attributes. All three cognitive board diversity qualities continue to be considerable and beneficial.

Furthermore, the findings corroborate the fundamental concept of agency theory that having a diverse board of directors reduces management’s opposing conduct and illogical choices and helps reduce business financial risks. The findings confirm the usefulness of cognitive board diversity in achieving intended financial performance (Talavera et al., 2018) and are consistent with earlier research (Anderson et al., 2011; Bernile et al., 2018; Ali et al., 2021b).

### Effect of cognitive board diversity on the financial distress likelihood in State-Owned Enterprises (SOEs) and Non-State-Owned Enterprises (NSOEs)

State ownership dominates Chinese firms and therefore is different from Western firms with minimum state ownership (Chen et al., 2016; Sarwar et al., 2020). Despite changes to limit state ownership, SOEs remain numerous and make up a large portion of Chinese listed firms (Usman et al., 2018; Jebran et al., 2020). According to property rights theory, private ownership is crucial for efficient and successful management supervision (Alchian, 1965). Therefore, based on this theory, the achievements of a company can be hampered by governmental ownership. For instance, SOEs are frequently considered to improve social welfare in contrast with shareholders’ wealth maximization viewpoint (Sarwar et al., 2020). Because SOEs and NSOEs are distinguishing characteristics of Chinese companies, we examine whether board diversity affects financial distress differently for SOEs and NSOEs.

State-Owned Enterprises (SOEs) and Non-State-Owned Enterprises (NSOEs) were then subsampled. Table 6 summarizes the findings. The results demonstrate significant positive coefficients on education diversity, financial expertise, and tenure diversity in all scenarios, namely in Columns 1, 2, and 3 for SOEs and Columns 4, 5, and 6 for NSOEs. The findings show that cognitive diversity reduces the chance of financial difficulty in both SOEs and NSOEs. On the other hand, the magnitude of the diversity coefficients is more significant in NSOEs, indicating that these diversity qualities are more helpful in reducing financial distress risk in NSOEs.

**TABLE 6:** Fixed effect regression results for SOEs and NSOEs.

Variables	SOEs				NSOEs			
		
	FD (1)	FD (2)	FD (3)	FD (4)	FD (5)	FD (6)	FD (7)	FD (8)
Edu_D	1.039***			0.986***	1.773***			1.565***
	−0.266			−0.261	−0.359			−0.356
Exp_D		0.690***		0.619***		0.758***		0.687***
		−0.092		−0.0911		−0.114		−0.114
Ten_D			0.949***	0.796***			1.155***	1.049***
			−0.117	−0.116			−0.147	−0.147
FS	−0.0254**	−0.0302**	−0.0576***	−0.0332**	−0.00799	−0.0117	−0.0321**	−0.0312**
	−0.0127	−0.0127	−0.0132	−0.0132	−0.0125	−0.0125	−0.0128	−0.0128
F_Lev	−0.469***	−0.442***	−0.451***	−0.484***	−0.149**	−0.142**	−0.126*	−0.09
	−0.0595	−0.0594	−0.0593	−0.0592	−0.0694	−0.0693	−0.0692	−0.069
F_Liq	0.161***	0.183***	0.155***	0.115***	0.0830**	0.0793**	0.0623*	0.0771**
	−0.0441	−0.0437	−0.0438	−0.0436	−0.0377	−0.0376	−0.0376	−0.0374
ROA	0.350***	0.369***	0.396***	0.342***	0.897***	0.899***	0.956***	0.952***
	−0.0927	−0.0922	−0.0921	−0.0912	−0.0973	−0.0971	−0.0972	−0.0966
BS	−0.303***	−0.296***	−0.235**	−0.215**	−0.186	−0.186	−0.126	−0.12
	−0.0913	−0.091	−0.0913	−0.0902	−0.117	−0.117	−0.117	−0.116
BI	0.218	0.261	0.271	0.228	−0.0793	−0.131	−0.134	−0.082
	−0.252	−0.251	−0.251	−0.248	−0.343	−0.342	−0.341	−0.339
Constant	1.277***	1.130***	1.829***	1.549***	0.369	0.324	0.864**	0.361
	−0.39	−0.389	−0.39	−0.39	−0.442	−0.44	−0.439	−0.441
Year dummies	Yes	Yes	Yes	Yes	Yes	Yes	Yes	Yes
Observations	7,072	7,072	7,072	7,072	5,293	5,293	5,293	5,293
Adjusted R^2^	0.03	0.036	0.038	0.062	0.03	0.034	0.038	0.049
Number of companies	861	861	861	861	659	659	659	659

***p < 0.01, **p < 0.05, *p < 0.1.

### Endogeneity problems

Furthermore, prior research implies an endogenous issue, namely simultaneity and causality, between board diversity and financial outcomes (Bernile et al., 2018; Yeh, 2018; Ali et al., 2021b). To overcome the possible endogenous problem, we used the system GMM regression described by Roodman (2009). In Table 7, a two-step GMM represents the association between cognitive board variety and financial distress probabilities. As shown in columns 1, 2, and 3 of Table 7, education board diversity and financial expertise positively influence the likelihood of financial distress for the sample as a whole, SOEs, and NSOEs, respectively, indicating that board diversity mitigates financial difficulties. While tenure diversity is insignificant, revealing the endogeneity problem for tenure diversity. In addition, the Arellano Bond (AR-2) and Hansen *p*-values showed that endogeneity was successfully handled. Table 7 confirms the conclusions of Table 5 in terms of educational diversity and financial expertise, indicating that these cognitive diversity traits are beneficial in reducing financial distress likelihood even after controlling for endogeneity.

**TABLE 7:** Endogeneity test using GMM techniques.

Dependent variable: Financial distress			

Variables	Full sample	SOEs	NSOEs
Lagged of dependent	0.281***	0.665***	0.736***
	−0.101	−0.169	−0.131
Edu_D	0.582**	1.104***	0.609**
	−0.281	−0.367	−0.278
Exp_D	0.969***	0.769***	0.888***
	−0.118	−0.16	−0.139
Ten_D	−0.0342	−0.331	−0.181
	−0.218	−0.233	−0.372
FS	−0.140***	−0.0372*	−0.0528**
	−0.0181	−0.0207	−0.0215
ROA	3.656***	0.355	0.642***
	−1.195	−0.227	−0.199
F_Lev	2.940***	−0.523***	−0.385***
	−0.338	−0.195	−0.147
F_Liq	0.507***	−0.206*	−0.0834
	−0.117	−0.122	−0.0619
BS	0.0298	−0.0991	0.103
	−0.0877	−0.139	−0.0983
BI	0.565**	−0.289	0.754**
	−0.249	−0.675	−0.336
Constant	0.849**	1.047*	0.376
	−0.381	−0.535	−0.553
Industry effect	Yes	Yes	Yes
Year effect	Yes	Yes	Yes
AR (1)-*p*-value	−7.71	−4.52	−6.88
	0	0	0
AR (2)-*p*-value	1.16	0.82	0.82
	0.245	0.38	0.41
Hansen’s J (*p*-value)	0.168	0.245	0.35
Observations	10,989	6,205	4,624
No. of companies	1,374	844	641

***p < 0.01, **p < 0.05, *p < 0.1.

## Conclusion, implications, and limitations

Specifically, we examine the association between financial distress of Chinese listed companies and characteristics of board diversity, such as education diversity, financial expertise, and tenure diversity. The study examines the proposed relationships using a fresh set of observations and improved methodological tools. The research findings are consistent with our theoretical framework, which is grounded on ideas from agency theory. This study empirically contributes to the works on management strategy and corporate governance in emerging markets. According to the study’s findings, cognitive board diversity attributes (educational diversity, financial expertise and tenure diversity) are advantageous in minimizing financial hardships. The study’s findings are also in line with board diversity literature, which has shown a negative relation between board diversity and business risk (Bernile et al., 2018; Ali et al., 2021a; Yousaf et al., 2021).

This study has implications for executives, investors, and practitioners. First, the findings demonstrate that directors with diverse educational backgrounds and financial understanding are important strategies for reducing financial distress risk. Second, the study’s results can aid shareholders and management in understanding how varied boards can shield businesses from financial difficulties and upsurge the company’s value. When appointing directors, they may consider cognitive board diversity to strengthen the board’s ability to make in-control decisions. Finally, our findings affect the nominating committee. The nomination committee has a vote on director recruitment and evaluations. The study suggests that the nomination committee must promote board education and diversity of financial experience.

The importance of diversity attributes for reducing the probability of financial distress has important policy implications. Regulators, for example, will be able to assess the effectiveness and process of diversity quotas properly. Furthermore, investors can better analyze the risks associated with various organizations based on their diverse arrangements. Firm and fund managers will be better able to implement appropriate governance systems and respond to changes in institutional environments that increasingly require diversity. Overall market quality, entrepreneurship, innovation, and equality can all be improved by encouraging efforts to capitalize on diversity.

Moreover, globally, authorities have mandated demographic board diversity, i.e., gender. However, the results of this study will assist policymakers and regulators in implementing board diversity laws by making them more aware of the benefits of cognitive board diversity. China is one of the countries that has not enacted laws governing board diversity. The Chinese legislative authorities will learn from this study that strict board diversity laws will assist firms in avoiding the danger of a financial crisis. In addition, developing board diversity guidelines that consider cognitive diversity will be a positive step for other economies that lack board diversity rules or focus solely on gender diversity. Furthermore, authorities may create separate corporate governance guidelines to address the financial dilemma, bearing in mind the board’s cognitive diversity.

Despite the study’s enormous output in the Chinese context, it has some drawbacks. First, the findings should be interpreted and extrapolated because this study’s sample was collected from China. Future research will expand on this approach by recruiting participants from other nations, revealing regional differences. Institutional determinants affect the link between board diversity and a firm’s economic performance, according to Ararat et al. (2018). Researchers could compare civil law and common law countries’ institutions in the future. Secondly, although we made every effort to resolve any endogeneity problems, the magnitude and direction of our coefficients might have caused problems. Other methods, such as difference-in-difference procedures, may be employed to handle potential endogeneity. Furthermore, Primary and secondary data triangulation can boost confidence. Finally, Future studies may examine how TMT features moderate this association.

## Data availability statement

The original contributions presented in this study are included in the article/supplementary material, further inquiries can be directed to the corresponding author.

## Author contributions

ShaA: writing – initial draft and analysis, visualization, and validation. ShoA: visualization, writing – literature review and editing, and methodology. JJ: review, editing, and supervision. MH: editing and methodology. GM: writing – literature review and editing. All authors contributed to the article and approved the submitted version.
